# USABILITY AND ACCEPTABILITY OF A PEER-DELIVERED TECHNOLOGY BASED MENTAL HEALTH INTERVENTION FOR FAMILY CAREGIVERS

**DOI:** 10.1093/geroni/igac059.2676

**Published:** 2022-12-20

**Authors:** Caroline Collins-Pisano, Amanda Leggett, Karen Fortuna

**Affiliations:** University of Colorado, Colorado Springs, Hanover, New Hampshire, United States; Wayne State University, Ypsilanti, Michigan, United States; Dartmouth College, Hanover, New Hampshire, United States

## Abstract

Family caregivers of people with dementia are critical to the quality of life of care recipients and the sustainability of healthcare systems but face increased risk of emotional distress and negative health outcomes. The purpose of this study was to examine the usability, acceptability, and preliminary effectiveness of a technology-based and caregiver-delivered peer support program, “Caregiver Remote Education and Support” (CARES) smartphone application and to identify barriers and facilitators to the ethical use of former caregivers as technology-based interventionists. Nine family caregivers of people with dementia received the CARES intervention and three former family caregivers of people with dementia were trained to deliver the CARES system. Quantitative data were collected at baseline and at the end of the two-week field usability study. Qualitative data were also collected at the end of the two-week field usability study. The pilot study demonstrated that a two-week, peer- delivered and technology supported mental health intervention designed to improve burden, stress, and strain levels was experienced by former and current family caregivers as usable, acceptable, and ethical. CARES was associated with non-statistically significant improvements in burden, stress, and strain levels. This pre/post field usability study demonstrated it is possible to train former family caregivers of people with dementia to use technology to deliver a mental health intervention to current caregivers. Future studies would benefit from a longer trial, a larger sample size, a randomized controlled design, and a control of covariables such as stages of dementia, years providing care, and the severity of dementia symptoms.

